# Myocardial and Atrial Strain Profiles in Pediatric Fontan Patients with Single Left Ventricle Using Two-Dimensional Speckle-Tracking Echocardiography: A Case–Control Study

**DOI:** 10.3390/jcm14228134

**Published:** 2025-11-17

**Authors:** Carmen Corina Șuteu, Andreea Cerghit-Paler, Liliana Gozar, Amalia Fagarasan, Nicola Suteu, Mihaela Iancu

**Affiliations:** 1 Department of Pediatrics III, “George Emil Palade” University of Medicine, Pharmacy, Science, and Technology of Târgu Mureș, 540139 Târgu-Mureș, Romania; 2 Department of Pediatric Cardiology, Emergency Institute for Cardiovascular Diseases and Transplantation, 540136 Târgu Mureş, Romania; 3 Department of Medical Informatics and Biostatistics, Iuliu Hațieganu University of Medicine and Pharmacy, 400012 Cluj-Napoca, Romania; miancu@umfcluj.ro

**Keywords:** Fontan circulation, single left ventricle morphology, speckle-tracking echocardiography

## Abstract

**Background/Objectives:** Children with single left ventricle (SLV) anatomy following Fontan palliation are at high risk for subclinical ventricular dysfunction, which may not be detected by conventional echocardiographic measures. Our objectives are as follows: (1) to assess myocardial and atrial strain profiles in pediatric Fontan patients with SLV using 2-dimensional speckle-tracking echocardiography (2D-STE), (2) to compare these findings with a healthy control group, (3) to investigate correlations with conventional echocardiographic and functional parameters. **Methods:** A single-center study of 66 pediatric patients, who underwent echocardiographic evaluation and a 6 min walk test (6 MWT). Conventional, 3D, and strain-based echocardiographic parameters were compared between groups. Correlations with clinical and functional indices were assessed using ANCOVA, analysis, generalized additive models, and Pearson’s correlation coefficient. **Results:** Fontan patients showed significantly reduced 6 MWT distances compared to controls (mean difference: 201.6 m, *p* < 0.0001). Post-test heart rate (HR) and oxygen saturation were significantly impaired (HR: 104.6 vs. 100.8 bpm, *p* = 0.0012; SaO_2_: 90.3% vs. 99.8%, *p* < 0.0001). Fontan patients showed statistically significant differences in nearly all the 2D parameters. Three-dimensional echocardiography revealed significantly lower left ventricular (LV) ejection fraction (*p* = 0.0020), higher end-diastolic (*p* = 0.0275) and end-systolic volumes (*p* = 0.0125) in the study group. Global longitudinal strain (LV_GLS) was reduced in Fontan patients compared to controls (*p* < 0.0001), with significant differences across nearly all LV segments. Left atrial (LA) reservoir and conduit strain were markedly decreased, while contractile strain remained similar. LV_GLS was negatively correlated with IVCT (r = −0.50, *p* = 0.0175). The LA reservoir strain (LASr_AC) significantly correlated with MAPSE (r = 0.43, *p* = 0.0461). **Conclusions:** In pediatric Fontan patients, myocardial and atrial strain imaging reveals subclinical dysfunction despite preserved conventional ejection fraction.

## 1. Introduction

The Fontan circulation represents the final palliative surgical procedure carried out on children with complex congenital heart defects (CHD) involving a single functional (SV) ventricle. Despite considerable progress in surgical techniques and perioperative care, Fontan physiopathology is associated with a high risk of hemodynamic complications and progressive cardiac dysfunction due to combined structural and functional factors [1,2]. Previous studies have demonstrated that chronic systemic venous hypertension, cyanosis, elevated pulmonary vascular resistance, and prolonged SV volume overload affect ventricular function [1]. The complex and variable anatomical structures, the abnormal ventricular outflow morphology, non-contractile septum, and the increased myocardial fibrosis also contribute to the impairment of the systolic-diastolic parameters of the SV [1,3]. Thus, an accurate and comprehensive assessment of SV function and systemic atrium is essential for early detection and intervention in structural or functional deterioration.

Although conventional 2-dimensional (2D) echocardiography has become the principal modality for assessing the univentricular heart, the evaluation of myocardial performance in SV patients is challenging due to load dependency and complex geometric morphology of the dominant ventricle [1,2,4,5]. All these factors limit the applicability of conventional 2D echocardiographic measures—such as ejection fraction (EF) by Simpson’s biplane method or M-mode shortening fraction—that are routinely used in biventricular hearts [6].

Three-dimensional (3D) echocardiography has proven accuracy in the assessment of ventricular volumes in anatomically normal hearts and correlates more closely with cardiac magnetic resonance (CMR) than 2D echocardiography [7,8]. Current recommendations suggest the use of 3D echocardiography where possible. Given its ability to provide more accurate volumetric and functional assessment, 3D echocardiography is increasingly recommended when available [9,10,11,12]. Recent advances in 3D echocardiography have enabled more accurate quantification of ventricular volumes and EF in CHD, including Fontan physiology, with several studies demonstrating its feasibility and improved geometric representation in SV patients [13,14]. Moreover, emerging 3D strain techniques have shown potential for detecting early ventricular dysfunction in pediatric Fontan cohorts; however, their widespread use remains limited by lower temporal resolution, technical demands, and variable image quality in this anatomically complex population. In contrast, 2D-STE remains more widely validated, accessible, and reproducible in pediatric SV follow-up, providing sensitive assessment of myocardial deformation even when acoustic windows are suboptimal. Therefore, our study utilizes 2D-STE to offer clinically applicable insight into myocardial performance while acknowledging the complementary role and future promise of advanced 3D-based imaging [13,14,15].

### Speckle-Tracking Echocardiography

Speckle-tracking echocardiography (STE) is an advanced non-invasive imaging method that allows detailed assessment of myocardial deformation. STE quantifies myocardial deformation by tracking acoustic markers within the myocardium, providing a sensitive, angle-independent assessment of ventricular function. Unlike conventional echocardiographic indices, STE can detect early subclinical dysfunction, which is particularly relevant in single-ventricle Fontan physiology characterized by chronic preload limitation and progressive myocardial remodeling. STE parameters include global and segmental longitudinal strain of the left ventricle, global longitudinal strain of the atrium, and the corresponding strain-rate parameters [16]. STE has been widely applied in the field of CHD. In univentricular hearts, STE has emerged as a valid, non-invasive, and convenient tool for assessing ventricular function. Published data demonstrate a strong correlation between myocardial strain measured by STE and findings from cardiac MRI and catheterization, further supporting its clinical applicability [1,17,18,19]. Due to its angle and geometric independence and good reproducibility, STE provides useful information on regional and global SV contractile function [2]. Moreover, strain indices are more sensitive than conventional echocardiography parameters, identifying subclinical ventricular dysfunction [2,19,20,21]. In the pediatric Fontan population, several studies have demonstrated the usefulness of STE in the early identification of ventricular and atrial dysfunction [4,19]. By enabling early detection of ventricular dysfunction, STE facilitates timely intervention before adverse cardiovascular events occur [19].

Longitudinal and circumferential strain analyses in comparative studies of single right ventricle (SRV) and single left ventricle (SLV) patients across various palliative surgical stages have revealed a progressive functional decline in SRV patients, suggesting early and intrinsic myocardial impairment that may affect long-term outcomes [22,23]. Moreover, strain parameters have been shown to correlate negatively with atrioventricular valve regurgitation [19]. Strain indices are now recognized as important prognostic markers in functionally single ventricle physiology [24,25]. In children with SLV physiology following Fontan palliation, Steflik et al., (2017) demonstrated that longitudinal strain indices correlate with pressure-volume loop parameters [18]. Additionally, regional strain and rotational analyses have proven essential in detecting subtle myocardial abnormalities characteristic of SLV physiology [26].

In Fontan patients, effective atrial function is essential for optimizing ventricular preload, given the increased dependence of diastolic filling on atrial systole [2,27]. Previous investigations have demonstrated that individuals with SV physiology exhibit increased atrial volumes and enhanced active atrial strain, accompanied by a significant reduction in reservoir and conduit atrial strain, when compared to subjects with biventricular cardiac anatomy [27,28]. In Fontan patients, global atrial strain does not correlate with invasively measured ventricular filling pressures [29]. However, atrial reservoir strain correlates positively with cardiac index and functional capacity (VO_2_), and its reduction has been associated with adverse outcomes, including mortality, transplantation, arrhythmias, and Fontan-specific complications such as protein-losing enteropathy and plastic bronchitis [26,27,30,31]. Studies by Lopez et al., (2018) and Khoo et al., (2013) further emphasized the relationship between atrial strain, rotational mechanics, and functional status in this population [26,27].

Given the ongoing variability in strain analysis methods, stemming from the lack of standardized reference values and limited inter-observer reproducibility, particularly in anatomically atypical hearts such as those seen in Fontan circulation, this study aims to comprehensively characterize myocardial and atrial strain profiles in pediatric Fontan patients with SLV anatomy using 2D STE, and to assess their correlations with functional status and conventional echocardiographic parameters.

## 2. Materials and Methods

### 2.1. Study Population

This is an observational single center study conducted between January 2025 and June 2025 at the Pediatric Cardiology Department of the Emergency Institute for Cardiovascular Diseases and Transplantation, Târgu-Mureș, a referral children’s pediatric cardiology hospital in Romania. The study enrolled 22 pediatric patients with SV physiology after the Fontan operation. To ensure a homogeneous cohort of subjects, pediatric patients with Fontan circulation whose SV had LV morphology were selected. Inclusion criteria were pediatric patients (children aged 4 to 17 years old) with completed Fontan circulation, absence of severe valvular dysfunction or associated structural anomalies that could interfere with STE analysis. Exclusion criteria were poor-quality echocardiographic images, unstable heart rhythm, recent surgical interventions (<3 months). Median Fontan follow-up was ~6 years (IQR 5–7 years), approximately 6.6 years on average.

As a control group, we included 44 healthy controls among similar age groups with no structural and functional heart defects, referred to the cardiology department for the detection of an innocent systolic murmur, with echocardiographic images available in the database.

The study protocol was approved by the local ethics committee, and informed consent was obtained from the legal guardians of all participants according to the principles of the Declaration of Helsinki.

Demographic data, clinical presentation, anatomical details, past interventions were extracted from medical records.

All patients underwent a routine physical examination. Clinical evaluation included anthropometric measurements (height and weight), peripheral oxygen saturation (SaO_2_), blood pressure, NYHA functional class (FC), and the 6 min walk test (6 MWT).

### 2.2. Echocardiography

All patients included in our study underwent detailed transthoracic echocardiographic evaluation using a Philips EPIQ CVx system (Philips, Andover, MA, USA), equipped with an X5-1 transducer and dedicated software for STE and 3D analysis using Philips Qlab 15.0 (material number 453562090801). The echocardiographic measurements were made according to the methods recommended by the American Society of Echocardiography (ASE) [32]. All images and measurements were obtained by a single investigator with over three years of experience in transthoracic echocardiography, including 2D STE and 3D modalities. The reliability of measurements was determined by the degree of endocardial border visualization in the included patients. Only images with clearly visualized endocardial borders in all segments were included in the analysis.

#### 2.2.1. Conventional 2D Echocardiographic Examination

**Figure 1:**
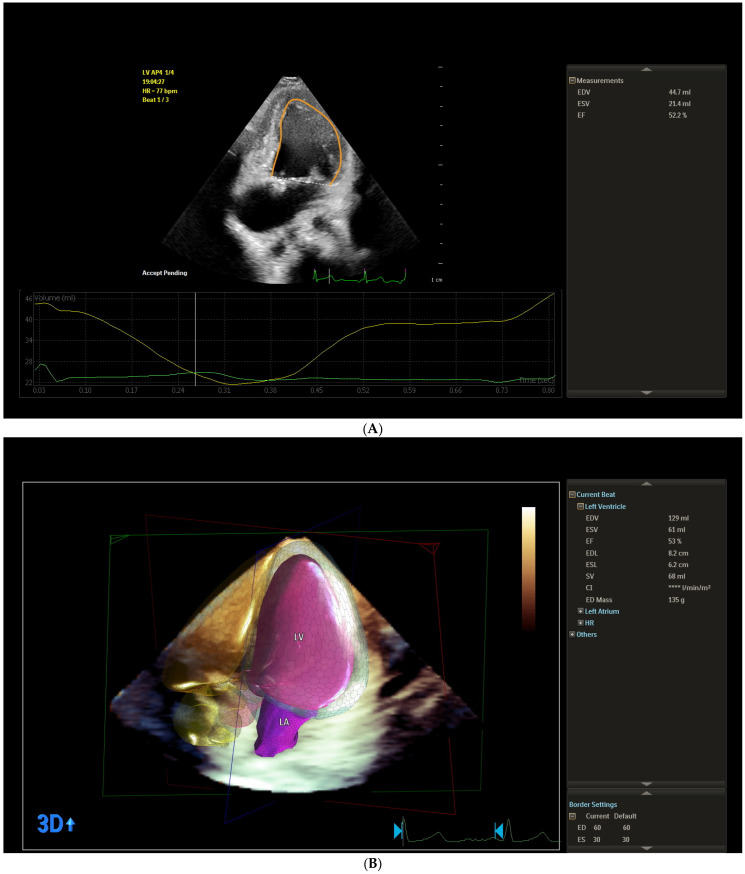

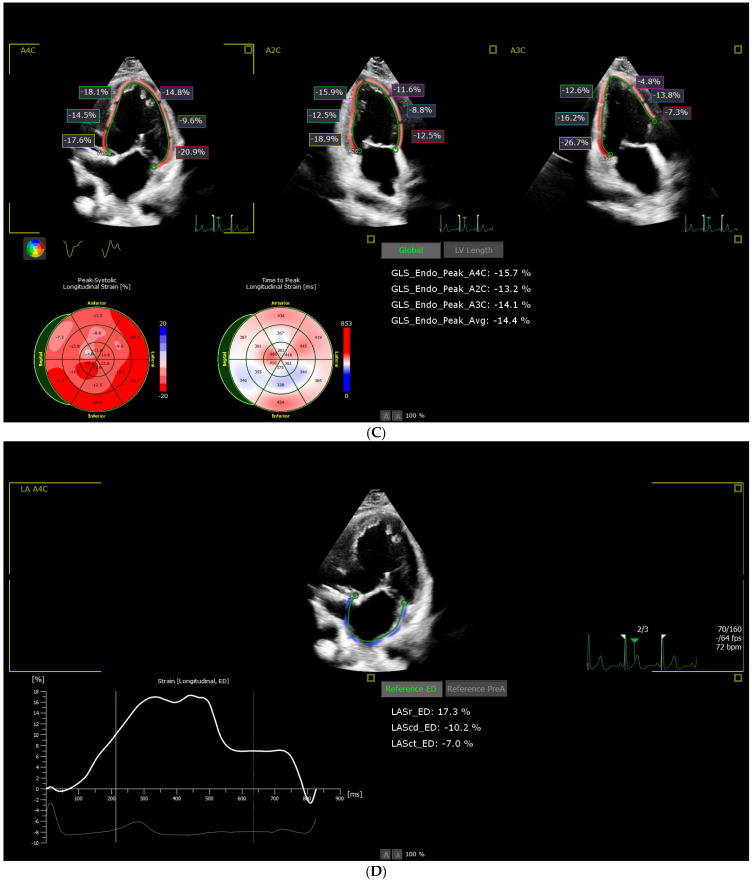
Representative example of (**A**) left ventricle ejection fraction measured by Simpson’s biplane method; (**B**) left ventricle ejection fraction measured by three-dimensional method; (**C**) left ventricular global longitudinal strain; (**D**) left atrial strain from the study sample.

Following anatomical assessment, conventional echocardiographic measurements were obtained in accordance with the guidelines of the ASE [32], interpreting the SV as functionally analogous to an LV. The echocardiographic protocol included lateral mitral annular plane systolic excursion by M-mode (MAPSE) and LV ejection fraction (EF) measured by Simpson’s biplane method (Figure 1A). For the Simpson’s biplane method, end-diastole was defined as the largest LV volume, and end-systole was defined as the smallest LV volume. Diastolic function of the functionally SLV was assessed using pulsed-wave Doppler by evaluating transmitral flow: peak mitral inflow velocity during early diastole (E_mi), peak mitral inflow velocity at atrial contraction (A_mi), LV isovolumic contraction time (IVCT), and LV isovolumic relaxation time (IVRT). Pulsed wave tissue Doppler was also used to measure peak annual systolic velocity (S_mi), mitral annular early diastolic velocity (E`_mi), and mitral annular late diastolic velocity (A`_mi).

#### 2.2.2. Advanced 3D Echocardiographic Examination

We assessed ventricular function using the 3D full volumetric method. The LV end-diastolic volume (mL) (LV_EDV), the LV end-systolic volume (mL) (LV_ESV), LV ejection fraction (%) (LV_EF (3D)), LV stroke volume (ml) (LV_SV), and end-diastolic LV mass (ED_mass) were measured. As part of the 3D acquisition protocol, an x-plane view of the ventricle was used to ensure that the relevant imaging planes were captured within the acquired volume. The rudimentary ventricle’s volume was not included in the univentricular volume measurement. Spatial and temporal resolutions were optimized by narrowing sector width, optimizing depth and focus. All image acquisitions were performed during end-expiratory breath-holding over 3 heartbeats to minimize motion artifacts. The mean frame rate used was 27 Hz, with a range from 21 to 59 Hz. The images were stored digitally for offline analysis. Volume analysis was performed using the 3D LV volume software package, Philips Qlab 15.0 (material number 453562090801), using the same definition for end-diastole and end-systole as for 2D acquisitions (Figure 1B).

#### 2.2.3. Speckle Tracking Acquisition and Analysis

Myocardial deformation was performed offline using the AutoStrain LV module of Philips QLab 15.0 (Philips, Andover, MA, USA). Appropriate phased-array transducers were selected according to patient size. Imaging settings were optimized to ensure high-quality acquisitions. Standard apical four-chamber (A4C), two-chamber (A2C), and three-chamber (A3C) views were acquired to ensure complete visualization of the systemic ventricle. Images were recorded during quiet respiration or breath-hold when feasible, with frame rates maintained between 60 and 90 frames per second. To enhance speckle-tracking accuracy, maximum imaging depth and minimal sector width were employed to improve spatial resolution and tracking fidelity. At least three consecutive cardiac cycles were digitally stored for offline analysis.

The endocardial borders of the SLV were manually delineated or automatically delineated at end-diastole, after which the software generated a region of interest (ROI) that included the full myocardial wall thickness. Manual adjustments were made if necessary to optimize tracking. Strain was calculated using 2D speckle tracking in the longitudinal plane. The reference point (REF) was set at end-diastole, corresponding to the onset of the QRS complex. Regional longitudinal strain (LS) was calculated automatically in 17 LV segments according to the model proposed by the ASE [32]: (basal inferoseptal-BIS (%), mid inferoseptal-MIS (%), apical inferoseptal-AIS (%), basal anterolateral-BAL (%), mid anterolateral-MAL (%), apical anterolateral-AAL (%), basal inferior-BI (%), mid inferior-MI (%), apical inferior-AI (%), basal anterior-BA (%), mid anterior-MA (%), apical anterior-AA (%), basal inferolateral-BIL (%), mid inferolateral-MIL (%), apical lateral-AL (%), basal anteroseptal-BAS (%), mid anteroseptal-MAS (%), apical anterior-AA (%). The software calculated LV_A4C_LS (%), LV_A2C_LS (%), and LV_A3C_LS (%). The primary strain parameter derived was global longitudinal strain (LV_GLS), computed as the average of peak negative strain values across all LV segments. Values were expressed as the percentage of myocardial shortening (Figure 1C).

Atrial deformation analysis was performed offline using the AutoStrain LA module of Philips QLab 15 (Philips, Andover, MA, USA) software. Standard A4C views were acquired, ensuring optimal visualization of the systemic atrium. The endocardial border of the systemic atrium was manually traced at end-systole. The software automatically generated a region of interest (ROI), which was manually adjusted to include the full myocardial thickness while excluding adjacent structures. Segmental tracking quality was verified visually, and segments with suboptimal tracking were excluded. The zero-strain reference point (REF) was set at end-diastole, corresponding to the onset of the QRS complex on the synchronized EKG trace. Atrial strain values were derived relative to this reference point. The following atrial strain parameters were calculated: reservoir strain (LASr)-peak positive strain during ventricular systole; conduit strain (LAScd)-passive emptying strain during early diastole; and contractile strain (LASct)-active atrial contraction strain in late diastole. Global strain values were automatically computed as the mean of all analyzable atrial segments included in the ROI. All values were expressed as percentages of myocardial deformation (Figure 1D).

### 2.3. Statistical Analysis

The statistical analysis was performed using R software version 4.5.1. (R Foundation for Statistical Computing, Vienna, Austria). Visual (quantile-quantile plots), descriptive (univariate skewness and kurtosis), and analytical methods (Shapiro–Wilk’s and Anderson–Darling tests) were used to assess whether the continuous variable followed a normal distribution. For normally distributed variables, descriptive measures were arithmetic mean (standard deviation, SD), while for log-normally distributed variables, geometric mean (geometric standard deviation, GSD) was used. Continuous variables that did not meet the assumption of normality or Log-normality were described using median and interquartile range (IQR: percentile 25th–percentile 75th). Qualitative variables were summarized using absolute and relative frequencies.

The independent-samples *t*-tests or chi-squared tests were used to compare demographic, body composition, and clinical characteristics between Fontan pediatric patients and the control group. Evolution of cardiorespiratory parameters assessed using 6 MWT by time and group was performed using a mixed-effects ANCOVA model.

Significant differences in distributions of conventional left ventricle echocardiographic parameters as well as two-dimensional echocardiographic segmental, global left ventricle longitudinal strain and left atrium strain in the Fontan and control groups were tested using independent-samples *t*-tests or Wilcoxon rank-sum tests with continuity correction. Associations were adjusted for body surface area (BSA) using ANCOVA, Welch ANCOVA, or generalized additive models (GAMs), as appropriate.

Correlation coefficients between conventional, echocardiographic global left ventricle longitudinal strain indices and left atrium strain indices were evaluated using Pearson’s correlation coefficient. Log-normally distributed variables were log-transformed (natural logarithm) prior to inclusion in the correlation analysis.

Intra-observer variability was evaluated using intraclass correlation coefficients (ICC) with a 95% confidence interval. Intra-observer variability was assessed based on a two-way mixed effects model, and estimated values of ICCs were interpreted as follows: an ICC ≥ 0.90 as excellent, 0.75 ≤ ICC < 0.90 as good reliability, 0.50 ≤ ICC < 0.75 as moderate, and ICC < 0.50 as poor reliability [33,34].

All two-sided statistical tests used a significance level (α) chosen at 0.05. In the present study, estimated significance level of *p* < 0.05 denoted a statistically significant result, while 0.05 ≤ *p* < 0.10 was considered marginally significant.

#### 2.3.1. Intra-Observer Variability

Ten randomly selected Fontan patients and ten randomly selected controls had atrial strain re-measured for intra-observer variability. Excellent intra-observer consistency was observed for all studied strain indices, with an intra-class correlation coefficient between 0.992 (95% CI: 0.967–0.998) and 0.999 (95% CI: 0.997–1) in both groups analyzed.

#### 2.3.2. Objectives

The objectives of the study are as follows: (i) to evaluate cardiorespiratory functional parameters using 6 MWT by time and group; (ii) to compare conventional 2D and 3D echocardiographic parameters of LV in controls and Fontan patients; (iii) to compare echocardiographic segmental and global strain parameters in controls and Fontan patients; and (iv) to investigate correlations between echocardiographic strain parameters and traditional indices.

## 3. Results

### 3.1. Description of Studied Groups

Twenty-two eligible Fontan patients (mean (SD): 10.59 (3.39) years, age values at assessment ranging from 4 to 17 years, and 44 controls with similar distribution of age and sex (*p* > 0.05, Table 1) were included in the current study. Median years from Fontan procedure was 5 years (IQR: [2.4, 9.75]). All patients in the Fontan group had an extracardiac Fontan. Sixteen (72.73%) Fontan patients had a moderate regurgitation, and six (27.27%) patients had a mild regurgitation.

### 3.2. Evolution of Cardiorespiratory Parameters Assessed Using Six-Minute Walk Test (6 MWT) by Time and Group

Means of 6 MWT distances decreased significantly from the control group to Fontan group.

(t(64) = 10.61, *p* < 0.0001, mean difference: 201.64 m, 95% CI for mean difference: 163.65–239.62). In Fontan group, means of 6 MWT distances decreased significantly between the Fontan patients classified as FC II and those classified as FC III [t(20) = 3.50, *p* = 0.0023, mean difference: 105.88 m, 95% CI for mean difference: 42,71–169.05).

After controlling for BSA as a covariate, the results of two-way mixed ANOVA analysis showed a statistically significant change in HR from pre-test to post-test between Fontan group and the control group (Table 2). The mean HR was significantly higher in the Fontan group at pre-test (estimated marginal means (EMM), 95% CI: 87.2, 83.2–91.2) compared to the control group (EMM, 95% CI: 77.3, 74.5–80.1) and higher in the Fontan group at post-test (EMM, 95% CI: 104.6, 100.7–108.6) compared to control group (EMM, 95% CI: 100.8, 98.0–103.6). The mean SaO_2_ was significantly lower in the Fontan group at pre-test (EMM, 95% CI: 92.1, 91.2–93.1) compared to the control group (EMM, 95% CI: 99.8, 99.1–100.5) and lower in the Fontan group at post-test (EMM, 95% CI: 90.3, 89.4–91.3) compared to control group (EMM, 95% CI: 99.8, 99.1–100.5).

**Figure 2:**
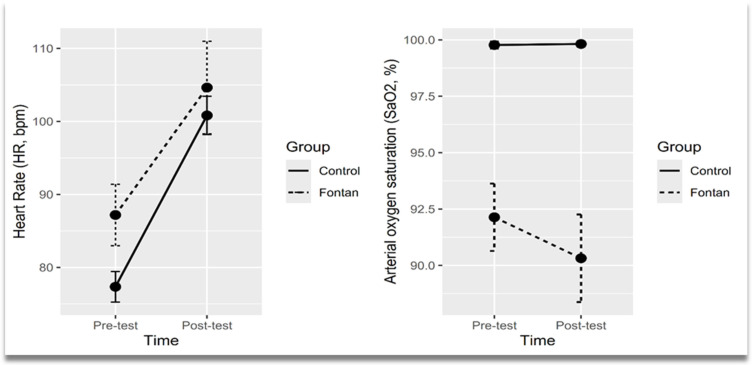
Profile plots showing means of heart rate and arterial oxygen saturation across time points by groups. Points represented mean values and error bars represented confidence interval (95% CI).

The effects of studied groups over time on cardiorespiratory parameters were depicted graphically in Figure 2. The profile plot showed a convergent trend between groups over time, indicating that the difference in HR between groups decreased over time. A slightly divergent pattern was noticed in the SaO_2_ profile plot (Figure 2).

### 3.3. Comparison of Conventional Left Ventricle 2D and 3D Echocardiographic Characteristics Between Fontan Group and Controls

Compared to control group, Fontan patients had statistically significant differences in nearly all the 2D studied echocardiographic characteristics after adjusting for BSA (Table 3). Among the 3D echocardiographic parameters, only EDV (adjusted *p*-value = 0.02754), ESV (adjusted *p*-value = 0.01246), ejection fraction (adjusted *p*-value = 0.0020), and ESL (adjusted *p*-value = 0.0280) showed significant differences after adjusting for BSA (Table 3).

### 3.4. Comparison of Two-Dimensional Echocardiographic Segmental and Global Left Ventricle Longitudinal Strain Indices in Controls and Fontan Patients

ANCOVA analysis revealed significant differences in segmental longitudinal strain indices between Fontan patients and the control group in all segments (Table 4), except for the mid anterolateral (F(1, 63) = 1.19, adjusted *p* = 0.2790) and basal inferior (F(1, 63) = 2.75, adjusted *p* = 0.1023) segments.

### 3.5. Corelations Between Conventional, Two-Dimensional Echocardiographic Global Left Ventricle Longitudinal and Left Atrium Strain Indices

Global left ventricle longitudinal strain indices were significantly linearly correlated with log-transformed LV IVCT in Fontan group, indicating that higher values of LV_GLS were associated with lower values of LV IVCT, but no significant linear correlation was observed between these two variables in the control group (r = −0.50, *p* = 0.0175). No other significant linear correlations were found between LV_GLS and conventional echocardiographic parameters in the Fontan group (Table 5).

LASr_ED indices were significantly linearly correlated with peak mitral inflow velocity at atrial contraction in Fontan group (r = 0.43, *p* = 0.0441), indicating that higher values of LASr_ED were associated with higher values of peak mitral inflow velocity at atrial contraction. LASCd_ED was negatively linearly correlated (r = −0.38) with MAPSE with marginal statistical significance (*p* = 0.0784), while LASr_AC was significantly linearly correlated with MAPSE (r = 0.43, *p* = 0.0461) in the Fontan group. We also noticed that LASr_Ac was positively correlated with peak mitral inflow velocity at atrial contraction with marginal significance (r = 0.41, *p* = 0.0598) in the Fontan group. LASct_AC was significantly linearly correlated with the ratio between early mitral inflow velocity and late mitral inflow velocity (r = 0.55, *p* = 0.0082) in the Fontan group (Table 6).

## 4. Discussion

Patients who have undergone Fontan palliation require lifelong surveillance and tailored clinical management due to their unique hemodynamic profile. In this study, we evaluate a cohort of Fontan patients and healthy controls to investigate early- to mid-term physiological and structural adaptations. Despite similar distributions of demographic characteristics, Fontan patients demonstrated higher resting heart rates and lower BMI and BMI Z-scores, findings that are consistent with prior studies and suggest altered autonomic regulation and impaired somatic growth in this population [35]. The median interval since Fontan completion was five years, capturing a clinically relevant period during which chronic hemodynamic load and systemic adaptations become apparent. All patients had undergone extracardiac conduit Fontan procedures, and a notable proportion exhibited moderate atrioventricular valve regurgitation-an established risk factor for adverse ventricular remodeling and long-term dysfunction [36]. These baseline characteristics reflect the multisystem impact of Fontan physiology and are essential for contextualizing the observed functional and echocardiographic findings in this cohort.

Patients with functional SV often remain asymptomatic due to self-imposed physical activity limitations, which can mask early manifestations of circulatory compromise. Consequently, cardiopulmonary exercise testing (CPET) is considered a standard component of functional assessment, as aerobic capacity parameters correlate with symptom severity and provide prognostic information [37,38]. The 6 MWT, a simple, non-invasive measure of submaximal exercise capacity, has gained widespread acceptance in the evaluation of children with CHD, including those with Fontan physiology. In this population, where preload dependency and absence of a subpulmonary ventricle limit cardiac output, the 6 MWT offers valuable insights into systemic perfusion and cardiovascular reserve. In our study, functional limitations were clearly evidenced by significantly reduced 6 MWT distances (mean difference: 201.64 m, *p* < 0.0001) and impaired chronotropic and oxygen saturation responses post-exercise in the Fontan group. Furthermore, a stratified analysis revealed that patients classified as FC III demonstrated significantly lower 6 MWT distances than those in FC II (mean difference: 105.88 m; t(20) = 3.50, *p* = 0.0023; 95% CI: 42.71–169.05), reinforcing the link between subjective functional status and objective exercise limitation. After adjusting for BSA, we also observed significantly higher heart rates in the Fontan group both at baseline (mean HR: 87.2 bpm vs. 77.3 bpm) and post-exercise (104.6 bpm vs. 100.8 bpm), indicating impaired chronotropic competence. Peripheral oxygen saturation (SaO_2_) was significantly reduced in Fontan patients both at baseline (92.1% vs. 99.8%) and following exertion (90.3% vs. 99.8%), reflecting impaired pulmonary vascular reserve and limited oxygen transport capacity. These findings are indicative of the chronic systemic venous hypertension and diminished pulmonary blood flow intrinsic to the Fontan circulation. Importantly, these clinical observations occurred despite largely preserved EF, reinforcing the disconnection between conventional measures and functional reserve in this population. These findings are consistent with previous reports demonstrating reduced 6 MWT performance, blunted heart rate responses, and desaturation during exertion in this population [28,30,31]. Given its simplicity, reproducibility, and correlation with CPET-derived indices, the 6 MWT remains a practical and valuable tool for longitudinal functional assessment and risk stratification in children with Fontan physiology [23,31].

Our study demonstrates that, after adjustment for BSA, Fontan patients exhibited significant differences in nearly all conventional 2D echocardiographic parameters compared to controls. Most notably, MAPSE and mitral annular velocities (E`_mi, A`_mi, S_mi) were reduced, consistent with impaired longitudinal systolic and diastolic function. These abnormalities are well-documented in SV physiology and likely reflect chronic myocardial remodeling and fibrotic changes [5,39]. In addition, diastolic time intervals, specifically IVCT and IVRT, were prolonged in the Fontan group, suggesting impaired myocardial relaxation and abnormal ventricular-arterial coupling, both of which have been associated with adverse clinical outcomes in this population [35,40]. The elevated E_mi/E`_mi ratio further supports this interpretation, indicating elevated filling pressures despite preserved conventional EF [35]. In the 3D echocardiographic analysis, Fontan patients demonstrated higher LV end-diastolic and end-systolic volumes, alongside a lower 3D-derived EF (LV_EF(3D)) (adjusted *p* = 0.0020), consistent with early systolic dysfunction. Importantly, this reduction in contractile performance was not consistently mirrored by 2D-derived EF values, underscoring the limitations of linear methods in detecting subtle ventricular compromise in Fontan physiology [39,41]. The observed differences in end-systolic length (ESL) may reflect altered ventricular geometry and adverse remodeling, changes that have been previously linked to long-term functional decline in Fontan survivors [36]. The absence of significant differences in LV_SV and LV_ED mass may represent either compensatory mechanisms or variability in volume loading and ventricular compliance across patients. Our study contributes to the growing evidence supporting the utility of 3D echocardiography, which offers more accurate volumetric assessment in Fontan patients. Taken together, these findings underscore the importance of a comprehensive echocardiographic evaluation, particularly the integration of 2D tissue Doppler imaging and 3D volumetric analysis, for the early detection of subclinical ventricular dysfunction in this population. This multiparametric approach may enhance risk stratification and support timely clinical decision-making in this vulnerable cohort [35,41,42,43].

The present study evaluates myocardial and atrial deformation in pediatric patients with single LV following Fontan palliation using 2D STE. Our findings demonstrated significant reductions in both global and segmental longitudinal strain indices when compared to healthy controls, reinforcing the clinical utility of myocardial deformation imaging in detecting early ventricular dysfunction in this anatomically and hemodynamically complex population.

A key finding was the marked reduction in LV_GLS in the Fontan cohort (mean −13.80%) compared to controls (−22.36%), despite a preserved median LV_EF (3D) above 50%. This observation underscores a key limitation of conventional EF in the evaluation of univentricular hearts. Due to altered ventricular geometry, load dependency, and non-standard myocardial fiber orientation, EF may fail to accurately reflect intrinsic myocardial contractility in this setting. In contrast, LV_GLS has emerged as a more sensitive and robust parameter for detecting subclinical systolic dysfunction, even in the presence of preserved EF. These findings are consistent with previous work by Liao et al., (2025), Kleitsioti et al., (2023), and Singh et al., (2010), who reported early myocardial impairment in Fontan patients attributable to chronic pressure and volume overload, systemic venous hypertension, and progressive myocardial fibrosis [1,2,19]. Importantly, the magnitude of LV_GLS reduction in our cohort (−13.80% vs. −22.36%) is consistent with values reported in other pediatric SLV studies, reinforcing its diagnostic and potential prognostic relevance [19,24]. Taken together, these results highlight the potential of GLS as an early and clinically meaningful biomarker of systolic dysfunction in the Fontan population, advocating for its routine incorporation into surveillance protocols.

In addition to the reduction in GLS, segmental analysis demonstrated impaired deformation across almost all LV regions, with the exception of the mid-anterolateral and basal-inferior segments. This heterogeneous strain pattern aligns with previous observations in univentricular physiology, reflecting regional differences in myocardial loading conditions, fiber orientation, and potential areas of localized fibrosis [3]. The greatest strain reductions occurred in the basal anteroseptal, mid-anterior, and apical inferoseptal segments, regions that may be particularly vulnerable to chronic hemodynamic stress, surgical manipulation during staged palliation, or intrinsic architectural alterations characteristic of single LV morphology. Similar segment-specific vulnerability has been highlighted in rotational and torsional mechanics studies, supporting the concept that remodeling in Fontan patients is not uniform but preferentially affects specific myocardial regions [26]. These findings emphasize the complementary value of segmental strain evaluation alongside global indices for early identification of regional functional impairment in this high-risk population.

Atrial strain analysis further substantiated the presence of diastolic dysfunction in the Fontan cohort. Both reservoir (LASr) and conduit (LAScd) strain values were significantly reduced compared to controls, while contractile strain (LASct) was relatively preserved. This pattern reflects impaired passive atrial filling and compliance, accompanied by a compensatory augmentation of atrial systole to support ventricular preload, an adaptive mechanism particularly relevant in the preload-dependent Fontan circulation. These findings are in agreement with previous studies by Khoo et al., (2013) and Li et al., (2014), which demonstrated increased atrial volumes and impaired reservoir and conduit function in patients with SV physiology, despite preserved atrial contraction [27,28]. The preservation of contractile strain in our cohort suggests a physiologic reliance on atrial systole to compensate for diminished ventricular filling during early diastole, underscoring the importance of atrioventricular synchrony in maintaining effective preload and cardiac output in Fontan patients. Importantly, atrial reservoir strain has emerged as a clinically relevant prognostic marker in the Fontan population. Prior studies have shown that lower LASr correlates with reduced cardiac index and diminished functional capacity and is independently associated with adverse outcomes such as heart failure, arrhythmias, and Fontan-specific complications including protein-losing enteropathy and plastic bronchitis [30,31]. Our findings further reinforce this association, highlighting the potential of atrial strain indices as non-invasive markers of diastolic performance and overall hemodynamic status in this population. Moreover, the results align with those of Lopez et al., (2018) [26], who identified abnormalities in atrial and ventricular rotational mechanics in post-Fontan patients, even when global longitudinal strain was preserved [32]. These observations suggest that regional deformation and atrial functional parameters may offer additional sensitivity in detecting subclinical myocardial dysfunction, which may be overlooked by traditional global systolic measures. As such, incorporation of atrial strain analysis into routine surveillance protocols may enhance early identification of patients at risk for clinical deterioration.

In this study, we investigate the associations between STE-derived LV and LA strain indices and conventional echocardiographic parameters in pediatric patients with SLV physiology post-Fontan procedure. Our findings demonstrate distinct patterns of correlation in the Fontan group compared to controls, highlighting the altered myocardial mechanics and atrioventricular interactions characteristic of Fontan circulation.

In the Fontan cohort, LV_GLS showed a significant inverse correlation with the log-transformed IVCT, suggesting that higher strain values were associated with improved systolic timing performance. This finding indicates that LV_GLS may be sensitive to subclinical alterations in electromechanical coupling in this unique physiological context and aligns with those of Steflik et al., (2017), who reported that strain parameters in SLV patients correlate with invasive pressure-volume loop measurements, reflecting their sensitivity to myocardial performance in this population [18]. In contrast, LV_GLS did not correlate significantly with conventional systolic parameters such as EF or MAPSE in Fontan patients, underscoring the added value of myocardial deformation imaging for evaluating systolic function in single-ventricle physiology. This lack of association is consistent with previous studies reporting that conventional volumetric indices are limited in univentricular hearts due to geometric distortion and preload dependency [1,2,6].

In healthy controls, LV_GLS demonstrated significant correlations with EF, MAPSE, and ESV, reflecting the expected interdependence between myocardial deformation and conventional systolic indices in structurally normal hearts. In contrast, the absence of these associations in the Fontan cohort likely reflects altered ventricular geometry, impaired myocardial mechanics, and the influence of abnormal loading conditions, such as diminished preload and increased afterload, which limit the sensitivity of standard volumetric measures. These divergent correlation patterns reinforce the diagnostic value of STE in Fontan physiology, where conventional metrics may underestimate subtle or early myocardial dysfunction [3,21].

Left atrial strain indices exhibited distinct correlation patterns with conventional echocardiographic parameters in the Fontan cohort, reflecting the altered atrioventricular dynamics of SV physiology. Notably, reservoir strain (LASr_ED) and booster pump strain (LASr_AC) were positively associated with peak mitral inflow velocity at atrial contraction (A_mi), suggesting that atrial contractile reserve is closely coupled with atrioventricular interaction in the absence of a subpulmonary ventricle. Additionally, LASr_AC showed a significant positive correlation with MAPSE, implicating ventricular longitudinal function as a determinant of atrial mechanical performance. In contrast, conduit strain (LAScd_ED) showed a negative trend with MAPSE, while contractile strain (LASct_AC) was significantly correlated with the E/A ratio, indicating a relationship between LA contraction and early diastolic LV function. These findings are consistent with prior studies demonstrating reduced reservoir and conduit strain but increased atrial contractile strain in Fontan patients, likely reflecting a compensatory mechanism for impaired passive ventricular filling [2,27,30]. Furthermore, reduced LA reservoir strain has been associated with adverse clinical outcomes, including arrhythmias, protein-losing enteropathy, and diminished functional capacity [30,31]. Collectively, our data support the role of speckle-tracking echocardiography-derived LA strain, particularly reservoir and contractile components, as sensitive indicators of functional compromise and potential prognostic markers in this high-risk population. In the control group, LA strain indices exhibited expected correlations with early diastolic parameters, including E wave and E/E` ratio, consistent with preserved diastolic function and the predominant role of passive ventricular filling in structurally normal hearts [28,31]. In contrast, the absence of such associations in the Fontan group highlights the fundamentally altered diastolic physiology resulting from surgical palliation. These contrasting patterns underscore the impaired atrioventricular interaction characteristic of Fontan circulation, wherein atrial performance becomes increasingly dependent on active systole and systolic function to compensate for reduced ventricular compliance and the lack of a subpulmonary pump.

Collectively, our findings underscore the diagnostic utility of STE in detecting subclinical myocardial and atrial dysfunction in pediatric patients with Fontan circulation. By providing sensitive, load-independent measures of myocardial deformation, STE offers incremental value over conventional echocardiography, particularly in anatomically complex SV hearts [19,20]. The observed differential correlations between LV and LA strain indices and conventional echocardiographic parameters support their potential role as early markers of functional decline. Specifically, indices such as LV_GLS and LASct_AC may serve as valuable targets for longitudinal surveillance and timely clinical intervention. Nonetheless, challenges remain regarding the standardization of strain analysis across diverse ventricular morphologies and loading conditions, as highlighted in recent studies [26,29].

Limitations: The relatively small sample size, single-center design, and cross-sectional nature limit generalizability and preclude longitudinal prognostication. The STE analysis was performed only in the longitudinal plane, and the lack of MRI correlation is also a limitation. Although minimized through a single experienced operator, inter-observer variability remains a potential limitation. Further multicenter longitudinal studies are needed to determine the prognostic value of deformation parameters and their potential to guide therapeutic strategies.

## 5. Conclusions

Our findings support the incorporation of STE into routine clinical surveillance of pediatric Fontan patients with SLV anatomy. STE enables sensitive, non-invasive detection of subclinical myocardial and atrial dysfunction, which may be overlooked by conventional echocardiographic measures. The integration of STE with conventional and 3D echocardiography may enhance early risk stratification and inform timely clinical interventions. For future prospective, multicenter studies with larger cohorts are warranted to validate these findings and establish the prognostic value of myocardial and atrial strain parameters in the long-term management of this high-risk population.

## Figures and Tables

**Table 1:** Demographic, body composition, and clinical characteristics in the Fontan and control groups.

Variable	Fontan Group (n = 22)	Control Group (n = 44)	*p*-Value
Age at assessment (years)	10.59 (3.39)	10.48 (3.49)	0.9042 ^(a)^
Age at Fontan procedure (years)	4 [4; 5]	NA	NA
Male sex (n, %)	14 (63.6)	28 (63.6)	1.000 ^(c)^
Heart rate (HR, bpm)	85.45 (9.71)	78.27 (6.82)	0.0009 * ^(a)^
Body mass index (BMI, kg/m^2^)	16.53 (3.53)	17.73 (2.30)	0.0173 * ^(a)^
BMI Z-scores	−0.91 (1.55)	0.02 (0.90)	0.0140 * ^(b)^
Body surface area (BSA, m^2^)	1.14 (0.35)	1.25 (0.32)	0.2319 ^(a)^
BSA Z-scores	−0.81 (1.11)	−0.05 (0.65)	0.0060 * ^(b)^
SBP (mmHg)	108.55 (8.91)	109.02 (8.30)	0.8305 ^(a)^
SBP Z-score	0.34 (0.67)	0.45 (0.59)	0.5078 ^(a)^
Initial diagnosis:			
DILV	7 (31.82)	NA	NA
TA + PS + VSD	9 (40.91)	NA	NA
Unbalanced AVC	6 (27.27)	NA	NA
Surgical procedures:			
Open fenestration	13 (59.09)	NA	NA
LPA dilation/stent	8 (36.36)	NA	NA
RPA dilation/stent	6 (27.27)	NA	NA
Closure of fenestration	2 (9.09)	NA	NA
Closure of veno-venous collaterals	4 (18.18)	NA	NA
Closure of arterio-venous collaterals	7 (31.82)	NA	NA
CoAo surgery	3 (13.64)	NA	NA

Data are expressed as mean (SD); SD: standard deviation or median (percentile 25th–percentile 75th) or absolute (relative frequencies, %), ^(a)^ Student’s *t*-test with equal variances was performed to compare the groups; ^(b)^ Student’s *t*-test with unequal variances was performed to compare the groups; NA: not applicable; ^(c)^ Chi-squared test; * statistical significance: *p* < 0.05; SBP-systolic blood pressure; DILV: double inlet left ventricle; TA: tricuspid atresia; PS: pulmonary stenosis; VSD: ventricular septal defect; AVC: atrioventricular canal; LPA: left pulmonary artery; RPA: right pulmonary artery; CoAo: coarctation of the aorta.

**Table 2:** Comparison of the pre-and post-test cardiorespiratory parameters of the 6 MWT test in the Fontan and the control groups.

Variable	Time	Fontan (n = 22) Mean (SD)	Control (n = 44) Mean (SD)	*p*-Value ^(b)^ Time ∗ Group	Fontan (n = 22) EMM (95% CI)	Control (n = 44) EMM (95% CI)	Partial η^2^
				0.0229 *			0.08
HR (bpm)	Pre-test	87.18 (9.49)	77.34 (6.87)		87.2 [83.2, 91.2]	77.3 [74.5, 80.1]	
	Post-test	104.64 (14.29)	100.82 (8.60)		104.6 [100.7, 108.6]	100.8 [98.0, 103.6]	
	*p*-Value ^(a)^	<0.0001 *	<0.0001 *				
				0.0012 *			0.15
SaO_2_ (%)	Pre-test	92.14 (3.37)	97.77 (0.52)		92.1 [91.2, 93.1]	99.8 [99.1, 100.5]	
	Post-test	90.32 (4.38)	99.82 (0.39)		90.3 [89.4, 91.3]	99.8 [99.1, 100.5]	
	*p*-Value ^(a)^	0.0299 *	0.3229				

Data are expressed as mean (SD); SD: standard deviation; ^(a)^ *p*-values obtained from paired *t*-test; ^(b)^ *p*-values for time and group interaction effect obtained from mixed ANOVA model controlled for covariate body surface area; EMM: estimated marginal mean of cardiorespiratory parameters controlling for body surface area as a covariate; partial η^2^ indicated the effect size for the interaction effect between group and time; HR: heart rate; SaO_2_: the oxygen saturation level measured by a pulse oximeter; * statistical significance: *p* < 0.05.

**Table 3:** Distributions of conventional left ventricle 2D and 3D echocardiographic parameters in the Fontan and the control groups.

Variable	Fontan Group (n = 22)	Control Group (n = 44)	*p*-Value	Adjusted *p*-Value
Left ventricular measurements				
MAPSE (mm)	12.38 (2.79)	17.24 (2.14)	<0.0001 * ^(a)^	<0.0001 *
EF (%)	52.09 (9.23)	59.75 (5.10)	0.00115 * ^(b)^	0.00005 *
E_mi (m/s)	0.79 (1.137)	0.96 (1.15)	0.01142 * ^(b)^	0.00145 *
A_mi (m/s)	0.68 (0.20)	0.62 (0.10)	0.1752 ^(b)^	0.0925
E_mi/A_mi	1.23 (0.49)	1.56 (0.18)	0.0045 * ^(b)^	0.0001 *
E`_mi (cm/s)	14.22 (1.41)	24.03 (1.20)	<0.0001 * ^(b)^	<0.0001 *
A`_mi (cm/s)	8.21 (1.45)	12.31 (1.28)	0.00001 * ^(b)^	0.0004 *
S_mi (cm/s)	7.33 (1.32)	13.02 (1.19)	<0.0001 * ^(b)^	<0.0001 *
E_mi/E`_mi	0.05 [0.03, 0.05]	0.03 [0.03, 0.04]	0.0057 * ^(c)^	0.0246 *
IVCT_mi (ms)	70.76 (1.25)	56.92 (1.20)	0.00006 * ^(b)^	0.00006 *
IVRT_mi (ms)	76.32 (16.75)	60.34 (10.26)	0.0003 * ^(b)^	0.00001 *
EDV (mL)	93.16 (1.40)	82.50 (1.42)	0.1800 ^(b)^	0.02754 *
ESV (mL)	42.56 (1.50)	35.00 (1.46)	0.0564 ^(a)^	0.01246 *
LV_EF (3D) (%)	52.50 [46.25, 60.00]	58.5 [55.00, 62.50]	0.0025 * ^(c)^	0.0020 *
EDL (mm)	71.15 (1.17)	73.94 (1.15)	0.3077 ^(a)^	0.1401
ESL (mm)	62.24 (1.16)	58.40 (1.14)	0.0827 ^(a)^	0.0280 *
LV_SV (mL)	50.18 (23.61)	52.84 (18.37)	0.6166 ^(a)^	0.4362
LV_ED mass (g)	77.75 (1.56)	83.12 (1.41)	0.5000 ^(a)^	0.2948

Data are expressed as arithmetic mean (SD); SD: standard deviation or geometric mean (GSD); GSD: geometric standard deviation or median (percentile 25th- percentile 75th); ^(a)^ Student’s *t*-test with equal variances was performed to compare the groups; ^(b)^ Student’s *t*-test with unequal variances was performed to compare the groups; ^(c)^ Wilcoxon rank-sum test with continuity correction was performed to compare the groups. * statistical significance: *p* < 0.05; adjusted *p*-values were obtained using ANCOVA (Analysis of covariance) or generalized additive models; MAPSE (mm): mitral annular plain systolic excursion; LV_EF (%): left ventricular ejection fraction; E_mi (m/s): peak mitral inflow velocity during early diastole (E wave); A_mi (m/s): peak mitral inflow velocity at atrial contraction (A wave); E_mi/A_mi: the ratio between early mitral inflow velocity and late mitral inflow velocity; E`_mi (cm/s): mitral annular early diastolic velocity (E`wave); A`_mi (cm/s): mitral annular late diastolic velocity (A`wave); S_mi (cm/s): mitral annular systolic velocity (S wave); E_mi/E`_mi: the ratio between early mitral inflow velocity and mitral annular early diastolic velocity (E/E’); IVCT_mi (ms): left ventricle isovolumic contraction time; IVRT_mi (ms): left ventricle isovolumic relaxation time; EDV (mL): left ventricle end-diastolic volume: ESV (mL) left ventricle end-systolic volume; EDL (mm): left ventricle end-diastolic longitudinal diameter; ESL (mm): left ventricle end-systolic longitudinal diameter; LV_SV (mL): left ventricle end-systolic volume, LV_ED mass (g): left ventricle end-diastolic mass; * statistical significance: *p* < 0.05.

**Table 4:** Distributions of echocardiographic global and segmental left ventricle longitudinal strain indices in the control groups and the Fontan patients.

Variable	Control Group (n = 44)	Fontan Group (n = 22)	*p*-Value	Adjusted *p*-Value
Left ventricular strain				
BIS (%)	−21.56 (5.22)	−14.13 (8.20)	0.0005 * ^(b)^	0.0003 *
MIS (%)	−21.96 (5.49)	−11.24 (6.05)	<0.0001 * ^(a)^	<0.0001 *
AIS (%)	−22.88 (5.80)	−11.65 (6.33)	<0.0001 * ^(a)^	<0.0001 *
BAL (%)	−26.18 (7.73)	−17.33 (9.62)	0.0001 * ^(a)^	0.0002 *
MAL (%)	−17.91 (6.84)	−16.10 (5.07)	0.2772 ^(a)^	0.2790
AAL (%)	−21.42 (6.34)	−15.79 (5.73)	0.0008 * ^(a)^	0.0006 *
BI (%)	−24.93 (8.24)	−21.50 (8.48)	0.1190 ^(a)^	0.1023
MI (%)	−18.15 (3.92)	−13.60 (5.24)	0.0002 * ^(a)^	0.0002 *
AI (%)	−21.06 (5.22)	−15.98 (8.04)	0.0114 * ^(b)^	0.0082 *
BA (%)	−24.00 (7.25)	−13.98 (8.67)	<0.0001 * ^(a)^	<0.0001 *
MA (%)	−20.83 (5.13)	−10.83 (5.87)	<0.0001 * ^(a)^	<0.0001 *
AA (%)	−19.73 (5.59)	−10.08 (6.24)	<0.0001 * ^(a)^	0.0005 *
BIL (%)	−28.11 (7.45)	−20.61 (9.36)	0.0008 * ^(a)^	0.0004 *
MIL (%)	−18.31 (4.25)	−12.93 (7.71)	0.0005 * ^(b)^	0.0047 *
AL (%)	−18.31 (4.25)	−12.93 (7.71)	0.0001 * ^(b)^	0.0001 *
BAS (%)	−22.07 (6.73)	−9.10 (6.31)	<0.0001 * ^(a)^	<0.0001 *
MAS (%)	−20.72 (4.58)	−8.26 (5.16)	<0.0001 * ^(a)^	<0.0001 *
AA (%)	−19.74 (5.59)	−10.08 (6.24)	<0.0001 * ^(a)^	<0.0001 *
LV_GLS	−22.36 (1.42)	−13.80 (2.48)	<0.0001 * ^(b)^	<0.0001 *
Left atrium strain				
LASr_ED (%)	49.26 (16.26)	26.01 (9.19)	<0.0001 * ^(b)^	<0.0001 *
LAScd_ED (%)	−36.93 (12.85)	−17.09 (8.74)	<0.0001 * ^(a)^	<0.0001 *
LASct_ED (%)	−12.21(6.47)	−10.50 (8.65)	0.3685 ^(a)^	0.3856
LASr_AC (%)	43.85 (12.46)	24.09 (8.46)	<0.0001 * ^(a)^	<0.0001 *
LAScd_AC (%)	−32.95 (10.52)	−15.95 (8.43)	<0.0001 * ^(a)^	<0.0001 *
LASct_AC (%)	−10.65 (5.05)	−8.21 (4.50)	0.0598 ^(a)^	0.0618

BIS: basal inferoseptal; MIS: mid inferoseptal; AIS: apical inferoseptal; BAL: basal anterolateral; MAL: mid anterolateral; AAL: apical anterolateral; BI: basal inferior; MI: mid inferior; AI: apical inferior; BA: basal anterior; MA: mid anterior; AA: apical anterior (assessed from 2-chamber view and 3-chamber view); BIL: basal inferolateral; MIL: mid inferolateral; AL apical lateral; BAS: basal anteroseptal; MAS: mid anteroseptal;r; LV_GLS: left ventricular global longitudinal strain; LASr_ED: left atrial reservoir strain by using the starting points of R-wave peak; LAScd_ED: left atrial conduit strain by using the starting points of R-wave peak; LASct_ED: left atrial contractile strain by using the starting points of R-wave peak; LASr_AC: left atrial reservoir strain by using the starting points of P-wave onset; LAScd_AC: left atrial conduit strain by using the starting points of P-wave onset; LASct_AC: left atrial contractile strain by using the starting points of P-wave onset; ^(a)^ Student’s *t*-test with equal variances was performed to compare the groups; ^(b)^ Student’s *t*-test with unequal variances was performed to compare the groups; * statistical significance: *p* < 0.05; adjusted *p*-values were obtained using ANCOVA (analysis of covariance) or Welch ANCOVA (models with body surface area as covariate).

**Table 5:** Correlations coefficients between conventional and echocardiographic global left ventricle longitudinal strain indices in the control groups and the Fontan patients.

	Fontan Group (n = 22)	Control Group (n = 44)
Correlation with LV_GLS	Correlation Estimate (*p*-Value)	Correlation Estimate (*p*-Value)
MAPSE (mm)	−0.11 (0.6258)	−0.33 (0.0300 *)
EF (%)	−0.27 (0.2324)	−0.42 (0.0044 *)
Log E_mi (m/s)	0.03 (0.9047)	−0.10 (0.5135)
A_mi (m/s)	0.08 (0.7353)	−0.06 (0.6761)
E_mi/A_mi	0.11 (0.6274)	−0.03 (0.8253)
Log E`_mi (cm/s)	−0.10 (0.6603)	0.15 (0.3192)
Log A`_mi (cm/s)	0.13 (0.5661)	0.08 (0.6005)
Log S_mi (cm/s)	0.08 (0.7356)	−0.27 (0.0801)
E_mi/E`_mi	0.12 (0.5907)	−0.09 (0.5615)
Log IVCT_mi (ms)	−0.50 (0.0175 *)	−0.07 (0.6550)
IVRT_mi (ms)	0.03 (0.8895)	0.23 (0.1314)
Log EDV (mL)	−0.12 (0.6027)	0.10 (0.5297)
Log ESV (mL)	−0.14 (0.5454)	0.35 (0.0201 *)
Log EDL (mm)	0.05 (0.8374)	0.11 (0.4612)
Log ESL (mm)	0.06 (0.7972)	0.08 (0.6085)
Log LV_SV (mL)	−0.10 (0.6719)	0.001 (0.9982)
Log LV_ED mass (g)	0.001 (0.9957)	0.14 (0.3754)

MAPSE (mm): mitral annular plain systolic excursion; LV_EF (%): left ventricular ejection fraction; E_mi (m/s): peak mitral inflow velocity during early diastole (E wave); A_mi (m/s): peak mitral inflow velocity at atrial contraction (A wave); E_mi/A_mi: the ratio between early mitral inflow velocity and late mitral inflow velocity; E`_mi (cm/s): mitral annular early diastolic velocity (E`wave); A`_mi (cm/s): mitral annular late diastolic velocity (A`wave); S_mi (cm/s): mitral annular systolic velocity (S wave); E_mi/E`_mi: the ratio between early mitral inflow velocity and mitral annular early diastolic velocity (E/E’); IVCT_mi (ms): left ventricle isovolumic contraction time; IVRT_mi (ms): left ventricle isovolumic relaxation time; EDV (mL): left ventricle end-diastolic volume; ESV (mL): left ventricle end-systolic volume; EDL (mm): left ventricle end-diastolic longitudinal diameter; ESL (mm): left ventricle end-systolic longitudinal diameter; LV_SV (mL): left ventricle stroke volume; LV_ED mass (g): left ventricle end-diastolic mass; Variables with the Log prefix are log-transformed using the natural logarithm; * statistical significance: *p* < 0.05.

**Table 6:** Correlations coefficients between conventional and echocardiographic left atrium strain indices in the control groups and the Fontan patients.

	Fontan Group (n = 22)	Control Group (n = 44)
	Correlation Estimate (*p*-Value)	Correlation Estimate (*p*-Value)
Correlation with LASr_ED		
MAPSE (mm)	0.40 (0.0634)	0.10 (0.5152)
EF (%)	−0.30 (0.1722)	0.17 (0.2628)
Log E_mi (m/s)	−0.04 (0.8545)	0.33 (0.0271 *)
A_mi (m/s)	0.43 (0.0441 *)	0.28 (0.0609)
E_mi/A_mi	−0.39 (0.0722)	0.03 (0.8591)
Log E`_mi (cm/s)	−0.15 (0.5090)	−0.07 (0.6682)
Log A`_mi (cm/s)	−0.06 (0.7973)	0.02 (0.8851)
Log S_mi (cm/s)	−0.13 (0.5791)	0.23 (0.1305)
E_mi/E`_mi	−0.12 (0.5843)	0.23 (0.1328)
Log IVCT_mi (ms)	0.16 (0.4782)	−0.01 (0.9322)
IVRT_mi (ms)	−0.19 (0.3983)	−0.15 (0.3371)
Log EDV (mL)	0.01 (0.9726)	0.07 (0.6713)
Log ESV (mL)	0.16 (0.4781)	−0.08 (0.6236)
Log EDL (mm)	0.05 (0.8361)	0.04 (0.7832)
Log ESL (mm)	0.26 (0.2474)	0.08 (0.6136)
Log LV_SV (mL)	−0.17 (0.4536)	0.15 (0.3233)
Log LV_ED mass (g)	0.09 (0.6880)	0.07 (0.6439)
Correlation with LAScd_ED		
MAPSE (mm)	−0.38 (0.0784)	−0.07 (0.6677)
EF (%)	0.27 (0.2268)	−0.15 (0.3298)
Log E_mi (m/s)	−0.18 (0.4189)	−0.35 (0.0189 *)
A_mi (m/s)	−0.21 (0.3373)	−0.25 (0.0964)
E_mi/A_mi	0.06 (0.7822)	−0.11 (0.4782)
Log E`_mi (cm/s)	0.16 (0.4748)	0.07 (0.6641)
Log A`_mi (cm/s)	0.30 (0.1683)	−0.04 (0.8095)
Log S_mi (cm/s)	0.24 (0.2853)	−0.12 (0.2621)
E_mi/E`_mi	0.02 (0.9454)	−0.14 (0.3561)
Log IVCT_mi (ms)	−0.20 (0.3643)	0.04 (0.8042)
IVRT_mi (ms)	0.13 (0.5534)	0.20 (0.1853)
Log EDV (mL)	−0.24 (0.2819)	−0.09 (0.5573)
Log ESV (mL)	−0.34 (0.1184)	0.09 (0.5723)
Log EDL (mm)	−0.21 (0.3453)	−0.06 (0.6908)
Log ESL (mm)	−0.37 (0.1144)	−0.09 (0.5799)
Log LV_SV (mL)	0.04 (0.8535)	−0.15 (0.3408)
Log LV_ED mass (g)	−0.02 (0.9317)	−0.03 (0.8327)
Correlation with LASct_ED		
MAPSE (mm)	0.19 (0.3920)	−0.12 (0.4532)
EF (%)	0.07 (0.7625)	−0.18 (0.2383)
Log E_mi (m/s)	0.32 (0.1468)	−0.17 (0.2625)
A_mi (m/s)	0.07 (0.7530)	−0.23 (0.1379)
E_mi/A_mi	0.12 (0.5922)	0.14 (0.3629)
Log E`_mi (cm/s)	0.07 (0.6795)	0.07 (0.6368)
Log A`_mi (cm/s)	−0.09 (0.6795)	−0.03 (0.8487)
Log S_mi (cm/s)	0.04 (0.8767)	−0.27 (0.0740)
E_mi/E`_mi	0.15 (0.5065)	−0.32 (0.0334 *)
Log IVCT_mi (ms)	0.21 (0.3590)	−0.04 (0.7822)
IVRT_mi (ms)	−0.01 (0.9757)	−0.001 (0.9843)
Log EDV (mL)	−0.09 (0.7069)	0.05 (0.7415)
Log ESV (mL)	−0.18 (0.4194)	0.05 (0.7568)
Log EDL (mm)	−0.07 (0.7452)	0.08 (0.6150)
Log ESL (mm)	0.01 (0.9807)	0.02 (0.9120)
Log LV_SV (mL)	−0.06 (0.7989)	−0.06 (0.6875)
Log LV_ED mass (g)	−0.11 (0.6189)	−0.09 (0.5546)
Correlation with LASr_AC		
MAPSE (mm)	0.43 (0.0461 *)	0.12 (0.4468)
EF (%)	−0.33 (0.1337)	0.18 (0.2345)
Log E_mi (m/s)	0.03 (0.8899)	0.35 (0.0201 *)
A_mi (m/s)	0.41 (0.0598)	0.27 (0.0811)
E_mi/A_mi	−0.32 (0.1424)	0.08 (0.6182)
Log E`_mi (cm/s)	−0.15 (0.5004)	−0.07 (0.6743)
Log A`_mi (cm/s)	−0.11 (0.6174)	0.01 (0.9305)
Log S_mi (cm/s)	−0.17 (0.4549)	0.20 (0.1857)
E_mi/E`_mi	−0.10 (0.6578)	0.21 (0.1686)
Log IVCT_mi (ms)	0.22 (0.3226)	−0.001 (0.9995)
IVRT_mi (ms)	−0.13 (0.5716)	−0.18 (0.2360)
Log EDV (mL)	0.04 (0.8468)	0.06 (0.6817)
Log ESV (mL)	0.20 (0.3637)	−0.10 (0.5108)
Log EDL (mm)	0.07 (0.7410)	0.04 (0.8009)
Log ESL (mm)	0.30 (0.1762)	0.06 (0.6819)
Log LV_SV (mL)	−0.16 (0.4709)	0.14 (0.3780)
Log LV_ED mass (g)	0.06 (0.8055)	0.03 (0.8291)
Correlation with LAScd_AC		
MAPSE (mm)	−0.38 (0.0784)	−0.07 (0.6680)
EF (%)	0.27 (0.2268)	−0.15 (0.32000
Log E_mi (m/s)	−0.22 (0.3257)	−0.35 (0.0185 *)
A_mi (m/s)	−0.20 (0.3844)	−0.22 (0.1503)
E_mi/A_mi	0.02 (0.9394)	−0.16 (0.2939)
Log E`_mi (cm/s)	0.15 (0.5057)	0.07 (0.6736)
Log A`_mi (cm/s)	0.32 (0.1427)	−0.03 (0.8264)
Log S_mi (cm/s)	0.25 (0.2651)	−0.14 (0.3552)
E_mi/E`_mi	0.003 (0.9888)	−0.11 (0.4928)
Log IVCT_mi (ms)	−0.23 (0.2983)	0.03 (0.8484)
IVRT_mi (ms)	0.10 (0.6560)	0.23 (0.1315)
Log EDV (mL)	−0.26 (0.2474)	−0.09 (0.5773)
Log ESV (mL)	−0.36 (0.1025)	0.11 (0.4821)
Log EDL (mm)	−0.23 (0.3136)	−0.05 (0.7360)
Log ESL (mm)	−0.36 (0.1029)	−0.07 (0.6638)
Log LV_SV (mL)	0.03 (0.8914)	−0.13 (0.4121)
Log LV_ED mass (g)	−0.002 (0.9924)	0.01 (0.9732)
Correlation with LASct_AC		
MAPSE (mm)	−0.07 (0.7584)	−0.15 (0.3349)
EF (%)	0.11 (0.6321)	−0.19 (0.2237)
Log E_mi (m/s)	0.36 (0.0970)	−0.18 (0.2409)
A_mi (m/s)	−0.37 (0.0940)	−0.22 (0.1484)
E_mi/A_mi	0.55 (0.0082 *)	0.12 (0.4195)
Log E`_mi (cm/s)	−0.02 (0.9405)	0.06 (0.7151)
Log A`_mi (cm/s)	−0.40 (0.0640)	−0.03 (0.8362)
Log S_mi (cm/s)	−0.17 (0.4545)	−0.25 (0.0969)
E_mi/E`_mi	0.19 (0.3982)	−0.32 (0.0315 *)
Log IVCT_mi (ms)	0.05 (0.8298)	−0.06 (0.7226)
IVRT_mi (ms)	0.05 (0.8305)	0.01 (0.9483)
Log EDV (mL)	0.39 (0.0708)	0.06 (0.6823)
Log ESV (mL)	0.28 (0.2055)	0.07 (0.6772)
Log EDL (mm)	0.28 (0.2053)	0.09 (0.5680)
Log ESL (mm)	0.11 (0.6257)	0.04 (0.8166)
Log LV_SV (mL)	0.24 (0.2887)	−0.04 (0.7810)
Log LV_ED mass (g)	−0.09 (0.6851)	−0.08 (0.6252)

MAPSE (mm): mitral annular plain systolic excursion; LV_EF (%): left ventricular ejection fraction; E_mi (m/s): peak mitral inflow velocity during early diastole (E wave); A_mi (m/s): peak mitral inflow velocity at atrial contraction (A wave); E_mi/A_mi: the ratio between early mitral inflow velocity and late mitral inflow velocity; E`_mi (cm/s): mitral annular early diastolic velocity (E`wave); A`_mi (cm/s): mitral annular late diastolic velocity (A`wave); S_mi (cm/s): mitral annular systolic velocity (S wave); E_mi/E`_mi: the ratio between early mitral inflow velocity and mitral annular early diastolic velocity (E/E’); IVCT_mi (ms): left ventricle isovolumic contraction time; IVRT_mi (ms): left ventricle isovolumic relaxation time; EDV (mL): left ventricle end-diastolic volume; ESV (mL): left ventricle end-systolic volume; EDL (mm): left ventricle end-diastolic longitudinal diameter; ESL (mm): left ventricle end-systolic longitudinal diameter; LV_SV (mL): left ventricle stroke volume; LV_ED mass (g): left ventricle end-diastolic mass; Variables with the Log prefix are log-transformed using the natural logarithm; * statistical significance: *p* < 0.05.

## Data Availability

The original contributions presented in this study are included in the article. Further inquiries can be directed to the corresponding author.

## Abbreviations

The following abbreviations are used in this manuscript:

6 MWT	Six-Minute Walk Test
A_mi	Peak mitral inflow velocity at atrial contraction (A wave)
A`_mi	Mitral annular late diastolic velocity (A` wave)
AA	Apical anterior segment (ASE model)
AAL	Apical anterolateral segment (ASE model)
AIS	Apical inferoseptal segment (ASE model)
AL	Apical lateral segment (ASE model)
BAL	Basal anterolateral segment (ASE model)
BAS	Basal anteroseptal segment (ASE model)
BA	Basal anterior segment (ASE model)
BI	Basal inferior segment (ASE model)
BIL	Basal inferolateral segment (ASE model)
BIS	Basal inferoseptal segment (ASE model)
MA	Mid anterior segment (ASE model)
MAL	Mid anterolateral segment (ASE model)
MAS	Mid anteroseptal segment (ASE model)
MI	Mid inferior segment (ASE model)
MIL	Mid inferolateral segment (ASE model)
ANCOVA	Analysis of Covariance
AVC	Atrioventricular Canal Defect
BSA	Body Surface Area
CHD	Congenital Heart Disease
CI	Confidence Interval
CoAo	Coarctation of the Aorta
CPET	Cardiopulmonary Exercise Testing
DBP	Diastolic Blood Pressure
DILV	Double Inlet Left Ventricle
E_mi	Peak mitral inflow velocity during early diastole (E wave)
E_mi/A_mi	Ratio between early and late mitral inflow velocities
E`_mi	Mitral annular early diastolic velocity (E` wave)
EDL	End-Diastolic Longitudinal Diameter
ED_mass	End-Diastolic Mass
EDV	End-Diastolic Volume
EF	Ejection Fraction
EMM	Estimated Marginal Mean
ESL	End-Systolic Longitudinal Diameter
ESV	End-Systolic Volume
FC	Functional Class (NYHA)
GAM	Generalized Additive Model
GSD	Geometric Standard Deviation
HR	Heart Rate
ICC	Intraclass Correlation Coefficient
IQR	Interquartile Range
IVCT_mi	Isovolumic Contraction Time
IVRT_mi	Isovolumic Relaxation Time
LAScd	Left Atrial Conduit Strain
LAScd_AC	Left Atrial Conduit Strain measured from P-wave onset
LAScd_ED	Left Atrial Conduit Strain measured from R-wave peak
LASct	Left Atrial Contractile Strain
LASct_AC	Left Atrial Contractile Strain measured from P-wave onset
LASct_ED	Left Atrial Contractile Strain measured from R-wave peak
LASr	Left Atrial Reservoir Strain
LASr_AC	Left Atrial Reservoir Strain measured from P-wave onset
LASr_ED	Left Atrial Reservoir Strain measured from R-wave peak
LPA	Left Pulmonary Artery
LS	Longitudinal Strain
LV	Left Ventricle
LV_ED mass	Left Ventricular End-Diastolic Mass
LV_EDV	Left Ventricular End-Diastolic Volume
LV_EF (3D)	Left Ventricular Ejection Fraction by 3D Echocardiography
LV_ESV	Left Ventricular End-Systolic Volume
LV_GLS	Left Ventricular Global Longitudinal Strain
LV_SV	Left Ventricular Stroke Volume
MAPSE	Mitral Annular Plane Systolic Excursion
NYHA	New York Heart Association
PS	Pulmonary Stenosis
RPA	Right Pulmonary Artery
RV	Right Ventricle
RVOT	Right Ventricular Outflow Tract
SaO_2_	Arterial Oxygen Saturation
SBP	Systolic Blood Pressure
SD	Standard Deviation
S_mi	Mitral annular systolic velocity (S wave)
SLV	Single Left Ventricle
SRV	Single Right Ventricle
STE	Speckle-Tracking Echocardiography
SV	Single Ventricle
TA	Tricuspid Atresia
VSD	Ventricular Septal Defect
VO_2_	Oxygen Consumption
